# Adjuvant Cytokine-Induced Killer Cell Therapy Improves Disease-Free and Overall Survival in Solitary and Nonmicrovascular Invasive Hepatocellular Carcinoma After Curative Resection: Erratum

**DOI:** 10.1097/01.md.0000482730.73797.3b

**Published:** 2016-04-18

In the article “Adjuvant Cytokine-Induced Killer Cell Therapy Improves Disease-Free and Overall Survival in Solitary and Nonmicrovascular Invasive Hepatocellular Carcinoma After Curative Resection”,^1^ which appeared in Volume 95, Issue 5 of *Medicine*, the symbols listed next to significant *P* values in Table 3 were originally given incorrectly. The article has since been corrected online.
